# Effects of dietary supplementation probiotic complex on growth performance, blood parameters, fecal harmful gas, and fecal microbiota in AA+ male broilers

**DOI:** 10.3389/fmicb.2022.1088179

**Published:** 2022-12-20

**Authors:** Qiangqiang Zou, Xinyan Fan, Yunhe Xu, Tieliang Wang, Desheng Li

**Affiliations:** College of Animal Husbandry and Veterinary Medicine, Jinzhou Medical University, Jinzhou, China

**Keywords:** probiotic complex, AA+ male broiler, growth performance, blood parameters, fecal harmful gas, fecal microbiota

## Abstract

In this study, *Bacillus subtilis*, *Clostridium butyricum* and *Enterococcus faecalis* were made into a probiotic complex (PC). The PC was supplemented in AA+ male broilers’ diets to investigate the effects of PC on broiler growth performance, carcass traits, blood indicators, harmful gas emissions in feces and microbiota. Three hundred and sixty 1-day-old AA+ male broilers with an average initial body weight (data) were randomly divided into 3 dietary treatments of 6 replicates each, with 20 birds per replicate. The control group (T0) was fed a basal diet, while the test groups (T1 and T2) were supplemented with 0.025 and 0.05% PC in the basal diet, respectively. The trail was 42 days. The results showed that the supplementation of 0.05% PC significantly (*p* < 0.05) improved average daily gain (ADG) and average daily feed intake (ADFI) of broilers from 22 to 42 days and 1–42 days. Compared to the control group, the breast rate was significantly higher in T2, and the thymic index was significantly higher than that in T1 treatment (*p* < 0.05). The addition of PC had no significant effects on antibody potency in broiler serum (*p* > 0.05), but significantly increased albumin and total protein content in serum (*p* < 0.05). The addition of PC reduced H_2_S and NH_3_ emissions in the feces; the levels of *Escherichia coli* and *Salmonella* in the feces were significantly reduced and the levels of *Lactobacillus* were increased. And the most significant results were achieved when PC was added at 0.05%. Correlation analysis showed a significant positive correlation (*p* < 0.05) between the levels of *E. coli* and *Salmonella* and the emissions of H_2_S and NH_3_. Conclusion: Dietary supplementation with a 0.05% probiotic complex could improve the growth performance of broilers and also reduced fecal H_2_S and NH_3_ emissions, as well as fecal levels of *E. coli* and *Salmonella*, and increased levels of *Lactobacillus*. Thus, PC made by *Bacillus subtilis*, *Clostridium butyricum* and *Enterococcus faecalis* is expected to be an alternative to antibiotics. And based on the results of this trial, the recommended dose for use in on-farm production was 0.05%.

## Introduction

Antibiotic promoters (AGP) had been widely used to improve growth performance and protect poultry from pathogens. However, many countries had banned or will soon ban the use of antibiotics in poultry feed due to the effects of antibiotic residues on human health (Aalaei et al., 2019). The search for alternatives to antibiotics was therefore an urgent task. Researchers were focused on finding alternatives to antibiotics that can improve growth performance and keep animals healthy (Khan et al., 2016). Green and healthy alternatives to antibiotics had been developed at this stage, such as probiotics, prebiotics, enzymes and acidifiers. Probiotics were often fed to poultry to potentially increase feed intake and nutrient absorption (Ghareeb et al., 2012). Many studies had demonstrated that probiotics can consistently induce positive effects on gastrointestinal morphology (Kim et al., 2021), nutrient absorption (Bai et al., 2017), antioxidant capacity (Shi et al., 2019), apoptosis (Wu et al., 2019), and immune response (Yahfoufi et al., 2018).

Among the many bacteria used as probiotics, the spore-forming genus *Bacillus* was favored by researchers and companies because its spores are resistant to harsh conditions and could be preserved for long periods of time at certain ambient temperatures (Chen et al., 2013; Wealleans et al., 2017). *Bacillus subtilis* was a facultative anaerobic or aerobic bacterium that can grow in the intestinal tract. In the animal intestine, *Bacillus subtilis* could produce substances such as antimicrobial peptides that enhance humoral and intestinal mucosal immunity (Lee et al., 2015). Most researchers had demonstrated that the addition of *Bacillus subtilis* to broiler diets can improve growth performance in broilers (Rhayat et al., 2017; Wealleans et al., 2017; Qiu et al., 2021; Zhang et al., 2021). *Clostridium butyricum* was an anaerobic bacterium that is widely found in the intestinal tract and feces of animals. Compared to *Lactobacillus* and *Bifidobacterium*, *C. butyricum* was more tolerant to lower pH, relatively high bile concentrations and higher temperatures (Zhang et al., 2016). Due to their probiotic properties, *C. butyricum* and *Bacillus subtilis* were widely used worldwide. Previous studies had shown that *C. butyricum* can significantly improve growth performance, nutrient metabolism and intestinal morphology in broilers (Takahashi et al., 2018; Huang et al., 2019; Molnár et al., 2020). *Enterococcus faecalis* was a *lactic acid bacterium* commonly found in the gastrointestinal tract of humans and animals (Cao et al., 2013). The addition of *E. faecalis* to the diet promoted the growth of beneficial bacteria in the gut and inhibits harmful bacteria, thus maintaining a healthy gut and enabling the animal to achieved higher growth performance (Adil and Magray, 2012; Ognik et al., 2017). *Enterococcus faecalis* had a strong colonization advantage over other probiotics and was therefore still widely used as a pharmaceutical and microbial additive (Al-Balawi and Morsy, 2020; Hanifeh et al., 2021; Zhang et al., 2022).

The birds gastrointestinal tract contains different microbial communities whose interactions had an important impact on the nutritional, immunological and physiological status of the host (Zhao et al., 2013). Therefore, supplementing the diet with antibiotic alternatives holds promise as a good option to improve overall gut health and immunity through the growth of specific microorganisms, thus improving the diet (Borda-Molina et al., 2018). In this regard, the addition of probiotics to the diet was seen as a promising alternative to antibiotics (Dev et al., 2020). Researchers had reported the effects of *B. subtilis*, *C. butyricum* and *E. faecalis* as single strains on broilers. However, based on the physiological characteristics and possible synergistic effects of these three probiotics, is it possible to speculate that their combined use in broiler diets will have a beneficial effect on broilers? Therefore, this trial investigated the supplementation of AA+ male broiler diets with a complex probiotic made from *B. subtilis*, *C. butyricum* and *E. faecalis* to study the effects on broiler growth performance, carcass traits, blood indicators, harmful gas emissions and fecal microbiota, and to look for possible links between fecal microbial content and apparent indicators.

## Materials and methods

### Ethics statement

The Animal Conservation and Utilization Committee of the JZMU approved the animal use agreement (No. JZMULL2021006).

### Probiotic sources

The main components of PC are *B. subtilis* (2 × 10^8^ cfu/g), *C. butyricum* (2 × 10^6^ cfu/g) and *E. faecalis* (1 × 10^6^ cfu/g).

### Animals and experimental design

Three hundred and sixty 1-day-old AA+ male broilers with similar body weight (44.77 ± 0.25) were selected and randomly divided into 3 treatment groups of 6 replicates each, with 20 chicks in each replicate. The control group (T0) was fed a basal diet, while the test groups (T1 and T2) were supplemented with 0.025 and 0.05% probiotic complex in the basal diet, respectively. The test period was 42 days.

### Animals feeding management

The base diet for the trial (Table 1) was configured with reference to the National Research Council (1994) version. Broilers were free to feed and drink. The test broilers house is a fully enclosed house with an automatic environmental control system to ensure optimum temperature and humidity. The broiler house has 48 pens to provide shelter for the broilers. Each pen holds ten broilers at a density of approximately 625 cm^2^ per broiler. Every two pens are 1 repeat. During rearing, the room temperature is 33°C for 1–3 days and then drops by 3°C per week to maintain a room temperature of around 22°C and humidity control of 40–70%. The lighting program on days 1–7 and 36–42 was 24 h per day throughout the trial period. The lighting program on days 8–30 provided 20 h per day and 4 h of darkness. After day 31, the darkness hours were gradually reduced.

**Table 1:** Composition and nutrient levels of the basal diet.

Items	Contents	
	Days 1–21	Days 22–42
Ingredients (%)^a^		
Corn	60.4	64.05
Soybean meal	34.4	30
CaHPO_4_	1.40	1.30
CaCO_3_	1.21	1.12
NaCl	0.25	0.25
Soybean oil	1.00	2.00
Choline chloride	0.05	0.05
Lysine	0.08	0.10
*DL*-Met	0.21	0.13
Premix	1	1
Total	100.00	100.00
Nutrient levels (%)^b^		
ME (MJ/Kg)	12.14	12.51
CP	21.17	19.24
Available phosphorus	0.38	0.36
Lys	1.29	1.15
Met	0.67	0.48
Met + Cys	1.00	0.72
Ca	0.92	0.87

a Each kg of premix provides: VA, 5,000 IU; VD, 10,000 IU; VE, 75.0 IU; VK_3_, 18.8 mg; VB_1_, 9.8 mg; VB_2_, 28.8 mg; VB_6_, 19.6 mg; VB_12_, 0.1 mg; Biotin, 2.5 mg; Folic Acid, 4.9 mg; D-Pantothenic acid, 58.8 mg; Nicotinic acid, 196.0 mg; Zn, 37.6 mg; Fe, 40.0 mg; Cu, 4.0 mg; Mn, 50.0 mg; I, 0.2 mg; Se, 0.2 mg.

b The nutrient levels were calculated values.

### Test indicator determination

#### Growth performance

Broilers were weighed at 1, 21, and 42 d. Feed intake was recorded in replicates throughout the trial and average daily feed intake (ADG), average daily weight gain (ADFI) and meat to feed ratio (F/G) were calculated.

#### Carcass traits

At the end of the 42-day trial, one broiler from each replicate was randomly selected and weighed live after a 12-h fast. Selected broilers were euthanized and carotid artery blood was collected for serum preparation. Weighing of post-mortem broiler carcasses, breast and drumsticks. Calculate carcass yield, breast rate and drumsticks rate. The heart, liver, spleen, thymus and bursa were isolated, the blood aspirated on filter paper, the surface fat and connective tissue removed and weighed. Calculate the organ index.

Organ index = fresh weight of organ (g)/live weight before slaughter (kg).

#### Blood indicators

On day 21 of the test, 2 ml of blood was collected from the lower wing vein of the broiler and used to prepare the serum. Blood samples collected from 42-day-old broilers were left at room temperature for 30 min and centrifuged at 1,200 r/min for 15 min to extract the supernatant. The potency of serum antibodies to Newcastle disease and avian influenza H9 is determined by a hemagglutination inhibition test. Serum levels of albumin (ALB), total protein (TP), globulin (GLOB), alanine transaminase (ALT), alkaline phosphatase (ALP) and glucose (GLU) were measured using a fully automated biochemical analyzer.

#### Harmful gas emissions

On day 42 of the trial, fresh chicken manure was collected from each replicate and ammonia and hydrogen sulfide emissions from the manure were determined using the method of Dang et al. (2020). The manure was placed in a 2 l plastic box with small holes attached to the side and fermented at room temperature (25°C) for 6, 12, 24, and 48 h. The air sample is then collected with a gas collection pump from above the small holes on either side. NH_3_ and H_2_S concentrations are measured in the range of 0.00–100.00 mg/m^3^.

#### Fecal microbiota

A 1 g sample of broiler manure from each replicate was collected weekly and transported on ice to the laboratory following the method of Dang et al. (2020). Each replicate of 1 g fecal sample was diluted and mixed with 9 ml of 1% peptone broth. The viable counts of *E. coli*, *Lactobacillus* and *Salmonella* in fecal samples were determined on McConkey agar plates, MRS agar plates and BS agar plates (in a 10 g/l peptide solution) in a biosafety cabinet. The microbial count is ultimately expressed as log_10_ colony forming units per gram of feces.

#### Data analysis

The data was designed using a completely randomized grouping design. Replicate pen serves as the experimental unit. Multiple comparisons of significant differences in means were performed using the one-way ANOVA LSD method in SPSS 25.0 and visualization was completed using Graphpad Prism 8. Results are expressed as mean and standard deviation, with *p* < 0.05 indicating a significant difference. The results of the correlation analysis are completed and displayed by R (V4.0.2). Pearson correlation analysis was performed using the R (V4.0.2) corrplot package for the 42 days indicators, which were significantly correlated at *p* < 0.05 when *R* > 0.6 or *R* < –0.6.

## Results

### Growth performance

**Figure 1:**
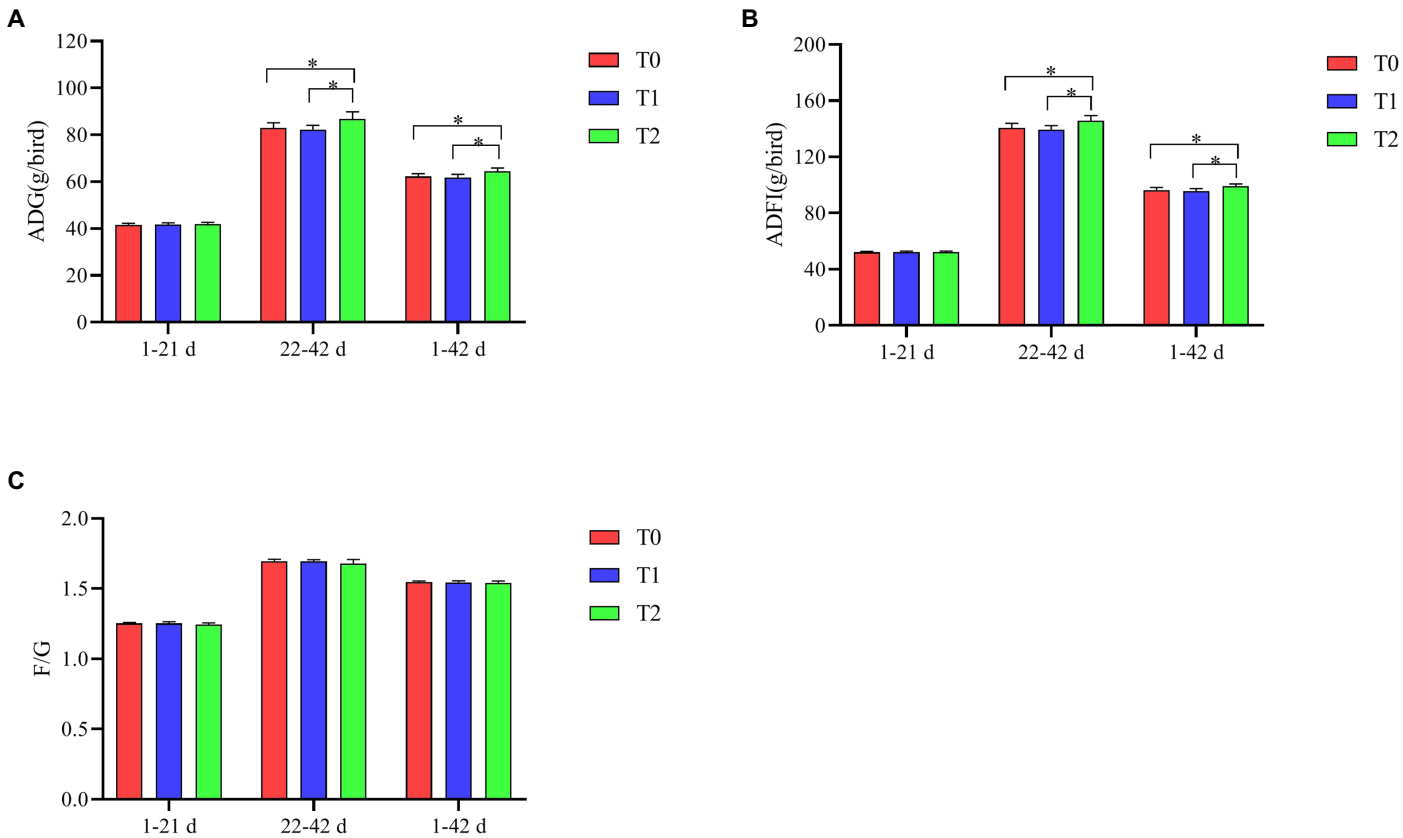
Effect of probiotic complex on growth performance of AA+ male broiler. **(A)** Average daily gain; **(B)** Average daily feed intake; and **(C)** Feed-to-weight ratio. T0: Feeding a basal diet; T1: Supplementation of the basal diet with 0.025% probiotic complex; T2: Supplementation of the basal diet with 0.05% probiotic complex. “*” means indicates a significant difference (*p* < 0.05). No “*” indicates that the difference is not significant (*p* > 0.05). The same as below.

As shown in Figure 1, ADG and ADFI were significantly higher at 22–42 d and 1–42 days in T2 compared to T0 and T1 (*p* < 0.05). The addition of the probiotic complex to the diet had no significant effect on F/G among the groups (*p* > 0.05).

### Carcass traits

**Figure 2:**
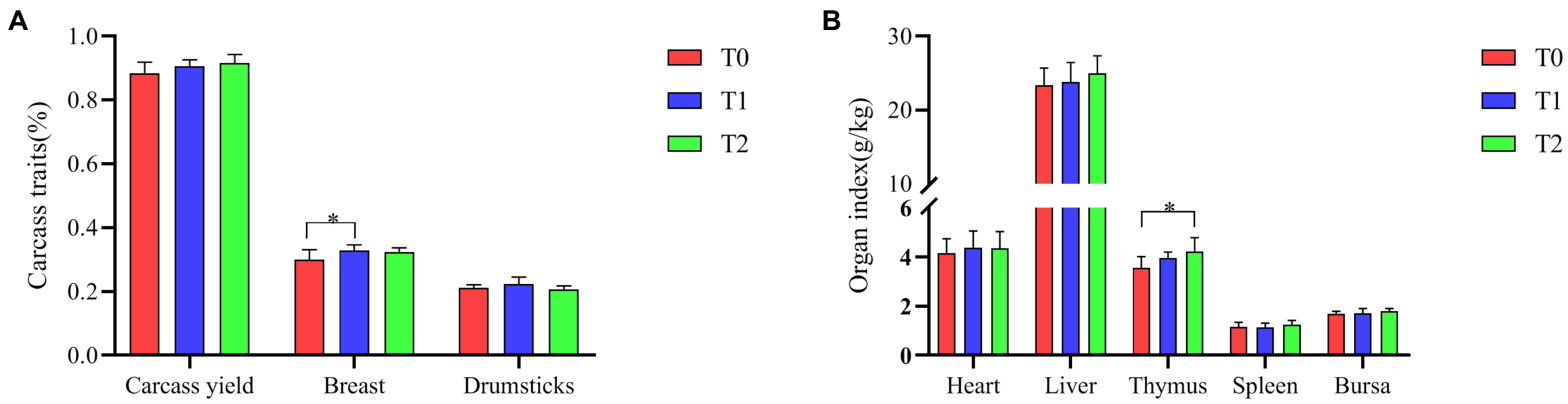
Effect of probiotic complex on carcass traits and organ indices of AA+ male broiler. **(A)** Carcass traits and **(B)** Organ index. T0: Feeding a basal diet; T1: Supplementation of the basal diet with 0.025% probiotic complex; T2: Supplementation of the basal diet with 0.05% probiotic complex.

As shown in Figure 2, the addition of the probiotic complex to the diet improved the carcass yield and liver index of broilers in the test groups (T1 and T2), but did not reach a significant difference (*p* > 0.05). Breast was significantly higher in T1 than in T0 (*p* < 0.05). Thymic index was significantly higher in T2 than in T0 (*p* < 0.05).

### Blood indicators

**Figure 3:**
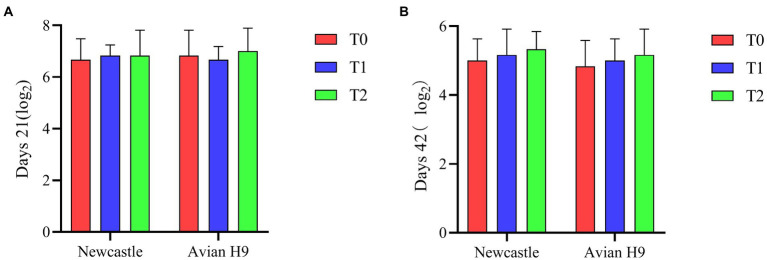
Effect of probiotic complex on serum antibody potency of AA+ male broiler. **(A)** Newcastle disease and Avian influenza H9 antibody potency in 21 days broiler sera. **(B)** Newcastle disease and Avian influenza H9 antibody potency in 42 days broiler sera. T0: Feeding a basal diet; T1: Supplementation of the basal diet with 0.025% probiotic complex; T2: Supplementation of the basal diet with 0.05% probiotic complex.

As shown in Figure 3, compared to the control group T0, supplementation of the diet with the probiotic complex increased the serum potency of Newcastle disease and avian influenza H9 antibodies in broiler in the test groups (T1 and T2), but did not reach a significant difference (*p* > 0.05).

**Figure 4:**
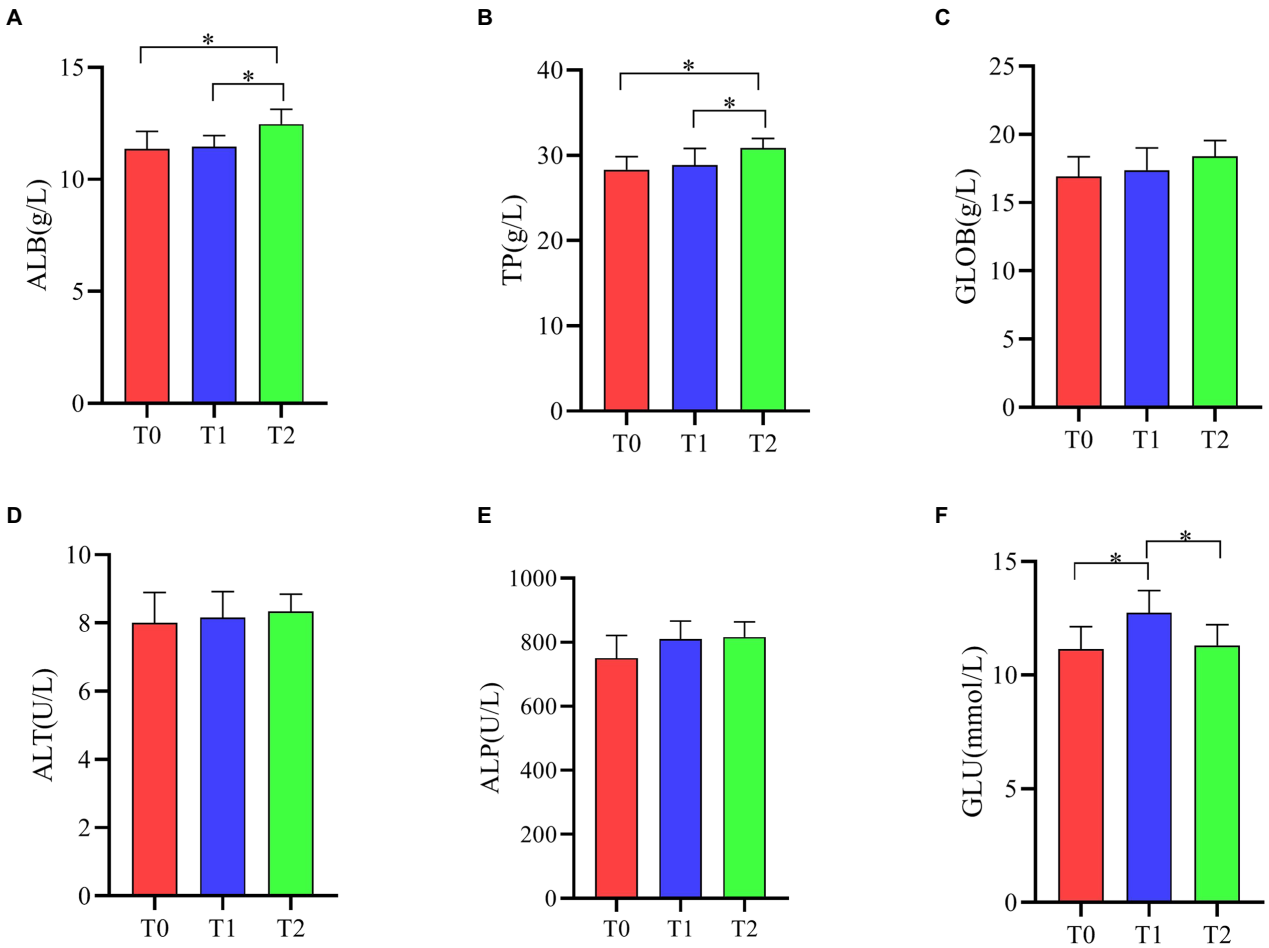
Effect of probiotic complex on serum biochemical parameters of AA+ male broiler. **(A)** Albumin; **(B)** Total protein; **(C)** Globulin; **(D)** Alanine transaminase; **(E)** Alkaline phosphatase; and **(F)** Glucose. T0: Feeding a basal diet; T1: Supplementation of the basal diet with 0.025% probiotic complex; T2: Supplementation of the basal diet with 0.05% probiotic complex.

As shown in Figure 4, ALB and TP levels were significantly increased in T2 serum compared to T0 and T1 (*p* < 0.05). GLU levels in serum were significantly higher in the T1 group than in the T0 and T2 groups (*p* < 0.05). Supplementation of the diet with the probiotic complex had no significant effect on the serum levels of GLOB, ALT and ALP in broiler (*p* > 0.05).

### Harmful gas emissions

**Figure 5:**
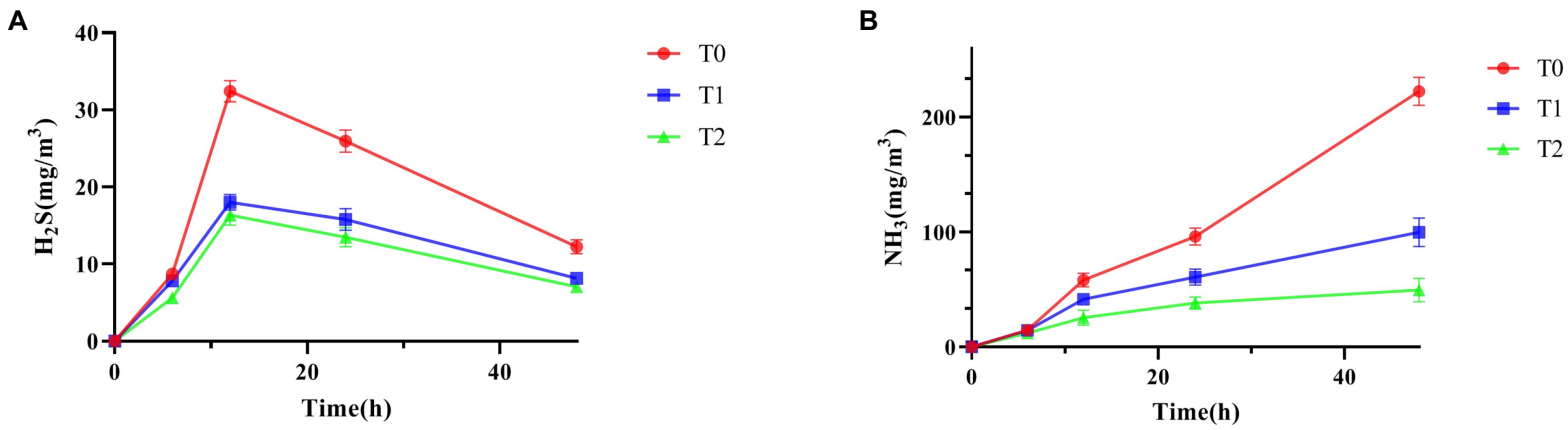
Effect of probiotic complex on harmful gas emissions of AA+ male broiler. **(A)** H_2_S; **(B)** NH_3_. T0: Feeding a basal diet; T1: Supplementation of the basal diet with 0.025% probiotic complex; T2: Supplementation of the basal diet with 0.05% probiotic complex.

As shown in Table 2 and Figure 5, supplementing the diet with a probiotic complex significantly reduced fecal H_2_S and NH_3_ emissions compared to the control group. And T2 was always kept the lowest level. As shown in Figure 5, it was clear that the emissions of H_2_S first increase and then decrease, while the emissions of NH_3_ showed a continuous increasing trend.

**Table 2:** Effect of probiotic complex on harmful gas emissions of AA+ male broiler.

Items	T0	T1	T2	*p*-Value
H_2_S				
6 h	8.71 ± 0.56^a^	7.82 ± 0.37^b^	5.60 ± 0.70^c^	<0.001
12 h	32.42 ± 1.38^a^	18.01 ± 1.01^b^	16.36 ± 1.31^c^	<0.001
24 h	25.96 ± 1.45^a^	15.79 ± 1.39^b^	13.48 ± 1.25^c^	<0.001
48 h	12.26 ± 0.91^a^	8.16 ± 0.54^b^	7.03 ± 0.79^c^	<0.001
NH_3_				
6 h	14.73 ± 1.34^a^	14.38 ± 1.14^a^	12.13 ± 1.23^b^	0.005
12 h	58.11 ± 5.82^a^	41.59 ± 5.04^b^	25.42 ± 6.61^c^	<0.001
24 h	96.8 ± 7.32^a^	60.80 ± 6.95^b^	38.21 ± 5.24^c^	<0.001
48 h	222.32 ± 12.24^a^	99.70 ± 12.40^b^	49.48 ± 10.17^c^	<0.001

H_2_S(mg/m^3^); NH_3_(mg/m^3^). ^a,b,c^Means in the same row with different superscripts are significantly (*p* < 0.05) different. The following table is the same. T0: Feeding a basal diet; T1: Supplementation of the basal diet with 0.025% probiotic complex; T2: Supplementation of the basal diet with 0.05% probiotic complex.

### Fecal microbiota

As shown in Table 3, the number of *Salmonella* in the feces of AA+ male broiler at 21 days was significantly lower in the T2 group than in the T0 group (*p* < 0.05). At 42 days, the number of *E. coli* in the feces of AA+ male broiler was significantly lower in groups T1 and T2 than in group T0 (*p* < 0.05), and there was no significant difference between groups T1 and T2 (*p* > 0.05). Supplementing the diet with a probiotic complex significantly reduced *Salmonella* levels in the feces of 42-day AA+ male broilers (*p* < 0.05) and had no significant effect on lactic acid bacteria levels in the feces (*p* > 0.05).

**Table 3:** The effect of probiotic complex on the microbial content of AA+ male broiler feces.

Items	T0	T1	T2	*p*-Value
Day 21				
*Escherichia coli*	5.76 ± 0.40	5.56 ± 0.21	5.41 ± 0.65	0.428
*Salmonella*	4.23 ± 0.10^a^	4.05 ± 0.15^ab^	3.97 ± 0.27^b^	0.066
*Lactobacillus*	8.42 ± 0.14	8.46 ± 0.32	8.72 ± 0.28	0.135
Day 42				
*E. coli*	6.33 ± 0.47^a^	5.90 ± 0.21^b^	5.62 ± 0.14^b^	0.004
*Salmonella*	5.49 ± 0.38^a^	4.25 ± 0.29^b^	3.52 ± 0.31^c^	<0.001
*Lactobacillus*	8.47 ± 0.28	8.77 ± 0.51	8.75 ± 0.39	0.385

*Escherichia coli* (log_10_ cfu/g); *Salmonella*(log_10_ cfu/g); *Lactobacillus*(log_10_ cfu/g). T0: Feeding a basal diet; T1: Supplementation of the basal diet with 0.025% probiotic complex; T2: Supplementation of the basal diet with 0.05% probiotic complex.

**Figure 6:**
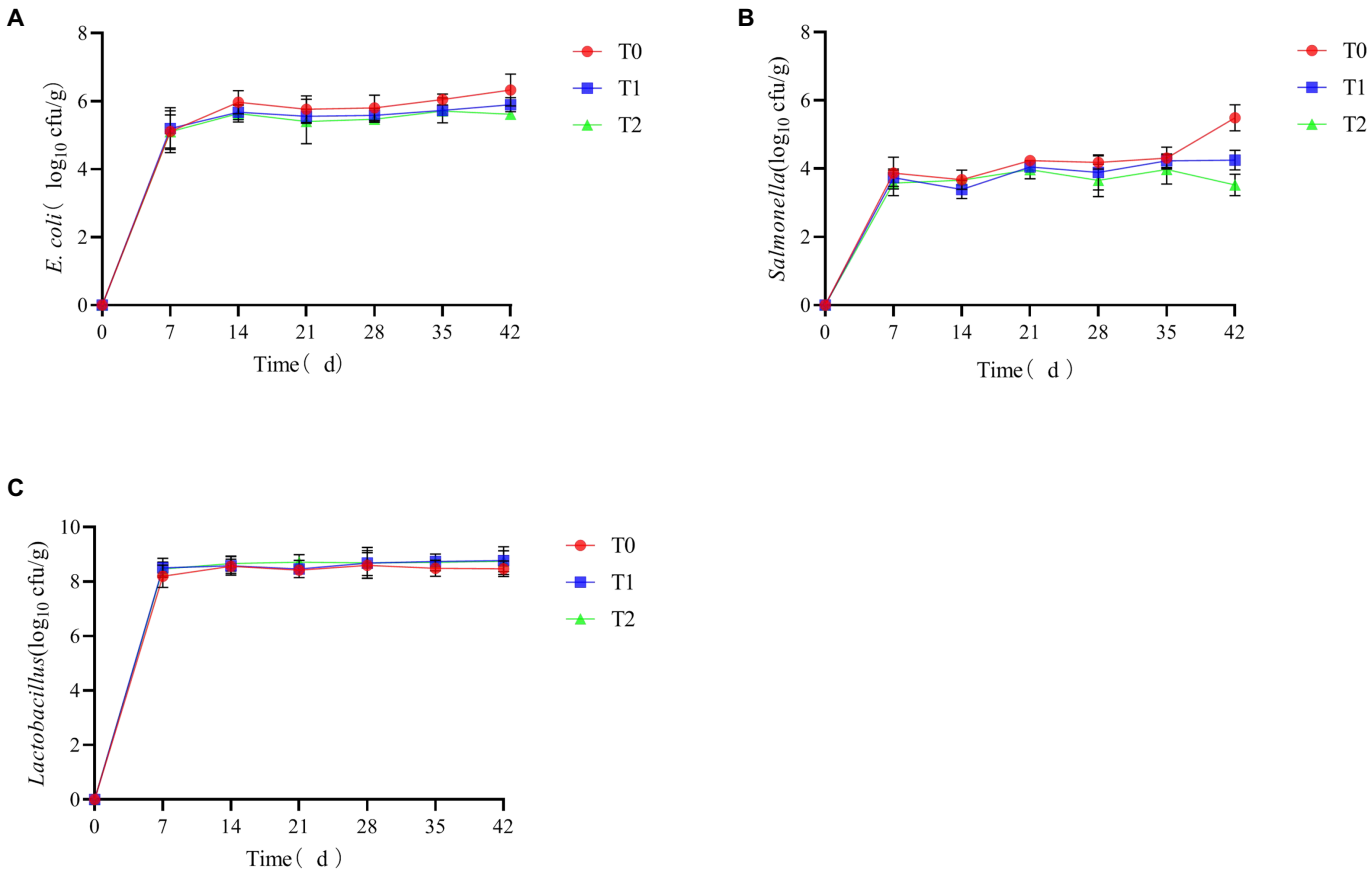
The effect of probiotic complex on the microbial content of AA+ male broiler feces. **(A)** *Escherichia coli*; **(B)** *Salmonella*; and **(C)** *Lactobacillus.* T0: Feeding a basal diet; T1: Supplementation of the basal diet with 0.025% probiotic complex; T2: Supplementation of the basal diet with 0.05% probiotic complex.

As shown in Figure 6, the levels of *E. coli* and *Salmonella* in the feces decreased throughout the breeding cycle after supplementation of the probiotic complex in the feed. In contrast, there was a slight increase in the levels of *Lactobacillus*.

### Correlation analysis of microbiological and surface indicators

**Figure 7:**
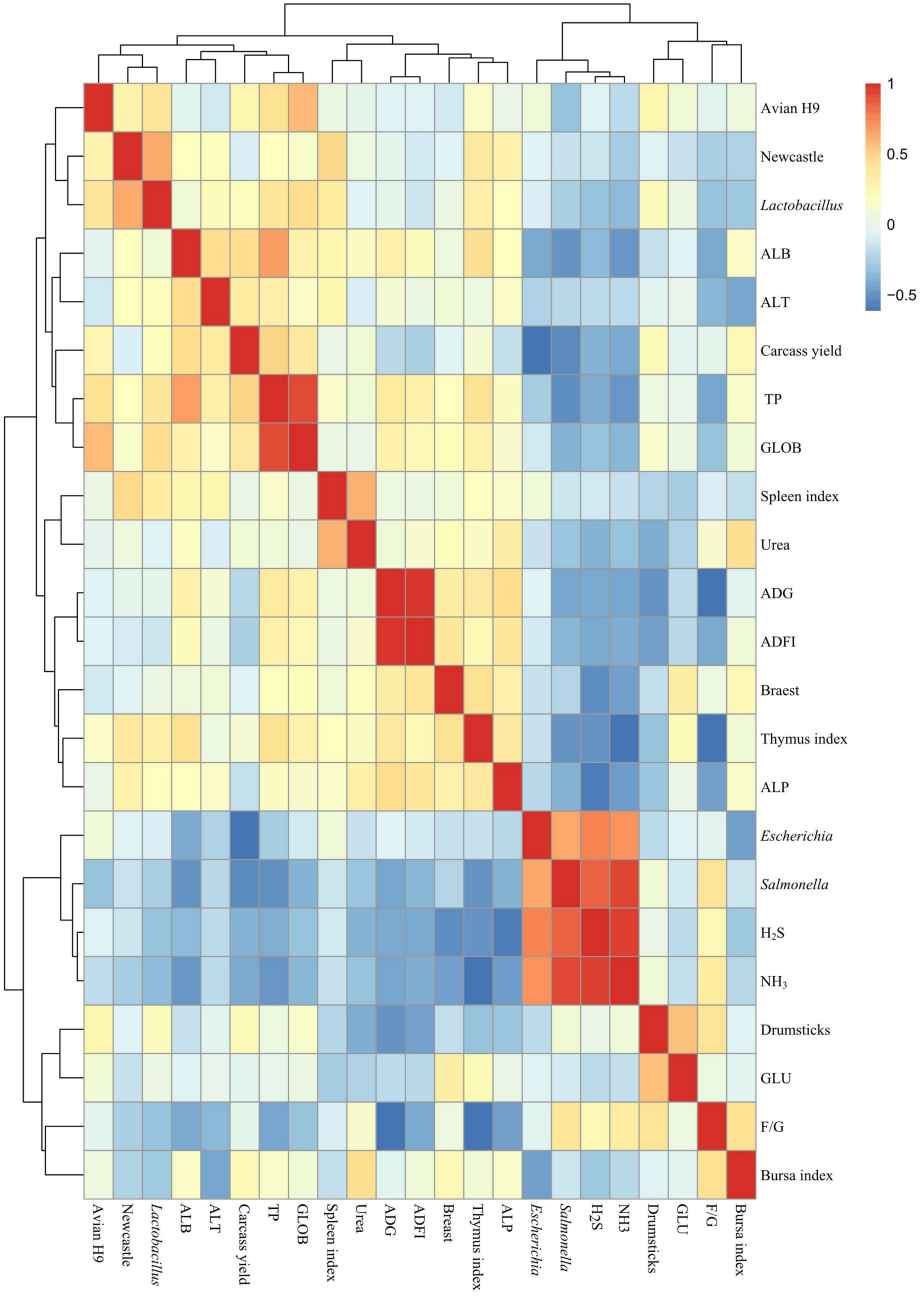
Correlation analysis of microbiological and surface indicators. T0: Feeding a basal diet; T1: Supplementation of the basal diet with 0.025% probiotic complex; T2: Supplementation of the basal diet with 0.05% probiotic complex.

As shown in Figure 7, carcass yield was significantly and negatively correlated with *E. coli* (*R* = −0.605). Thymus index was negatively and significantly correlated with NH_3_ emissions (*R* = −0.605). Newcastle disease antibody potency was significantly and positively correlated with *Lactobacillus* (*R* = 0.622). TP was significantly and positively correlated with ALB (*R* = 0.665) and GLOB (*R* = 0.912). *E. coli* was positively and significantly correlated with *Salmonella* (*R* = 0.643) and positively and significantly correlated with H_2_S (*R* = 0.745) and NH_3_ (*R* = 0.695). *Salmonella* was positively and significantly correlated with H_2_S (*R* = 0.840), NH_3_ (*R* = 0.934). H_2_S and NH_3_ (*R* = 0.943) were significantly and positively correlated.

## Discussion

Average daily gain, ADFI and F/G, as important indicators for evaluating growth performance, had a direct impact on the economic performance of the farming industry. Supplementation of broiler diets with single or multiple probiotics had been reported to promote growth performance and health by regulating the intestinal microbiota, improving digest and enhancing immune regulation (Yang et al., 2012). The results of this study showed that the addition of 0.05% PC, including significantly increased ADG and ADFI in broilers. The addition of 0.025% of probiotic complex also improved growth performance compared to the control, but not to a significant level. Previous studies have reported that the synergistic effects of combined probiotics are superior to those of single probiotics (Javandel et al., 2019; Salehizadeh et al., 2019). Wang et al. (2022) reported that the addition of *Bacillus* sp. *KC1* to the diet improved the growth performance and body weight of broilers. Zhang et al. (2021) also confirmed that the improvement in growth performance of broiler chickens by *B. subtilis* may be associated with improved microecology in the intestinal tract. Zeng et al. (2021) found that the addition of a mixture of *C. butyricum*, *B. subtilis* and *B. licheniformis* to broiler diets increased the body weight and average body weight of broilers. Shehata et al. (2020) added *Enterococcus faecalis-1* to broiler diets and found that *E. faecalis* improved growth performance in broilers. The results of this trial were similar to those previously reported above. In this trial, it was found that the addition of *B. subtilis*, *C. butyricum* and *E. faecalis* as the main components of the probiotic complex to the diet mainly improved growth performance during the late growth period (22–42 days) and indirectly during the full growth period (1–42 days). This may be related to the accumulation of probiotics in the intestinal tract. Probiotics can only have a positive effect on the organism when they have accumulated in the intestinal tract in certain quantities.

Carcass yield, breast rates and drumsticks rates reflect, to some extent, the meat production performance of broilers and, indirectly, their growth performance. The results of this study showed that the addition of 0.025% of a probiotic complex to the diet significantly increased the breast rates compared to the control group. However, the addition of a probiotic complex had no effect on carcass yield and drumsticks rates, which was consistent with the findings of Yadav et al. (2018) and Tang et al. (2021). The organ index was an important indicator of organ development in broilers. The results of this study showed that the addition of probiotic complexes to the diet had no effect on the cardiac index, liver index, spleen index and bursal index of broilers. However, the broiler thymus index increased significantly when added at 0.05%. The thymus was the lymphatic organ of the broiler. The increased weight of the lymphoid organs may indicate that treated broilers had acquired a higher level of immunity (Chen et al., 2013; Pournazari et al., 2017). Chen et al. (2013) reported that an increase in relative organ weight may coincide with an increase in lymphocyte concentration. Al-Sagan et al. (2020) reported a significant difference in the effect of probiotic addition on 29.3% of lymphoid organs associated with immune response compared to the supplemented group. In this trial, the addition of *B. subtilis*, *C. butyricum* and *E. faecalis* increased the thymus weight of broilers, which also suggests that the addition of probiotic complex could potentially improve the immunity of the broiler organism and enhance its ability to produce humoral and cellular immunity in response to antigenic stimuli.

Blood indicators were used for assessment to determine the quality of the feed/additive tested, reflecting the physiological state of the animal (Ogbuewu et al., 2015). The spleen, a lymphoid organ in broilers, activated the proliferation and differentiation of T and B cells and was the main site of antibody production. The results of this study showed that the addition of a probiotic complex to the diet increased antibody levels in broiler serum at 21 and 42 days, but did not reach a significant difference. Salehizadeh et al. (2019) reported that complex probiotics were effective in increasing Newcastle disease antibody levels in broilers. He et al. (2019) showed that *B. subtilis* had a positive effect on increasing Newcastle disease antibody levels in the blood of broiler. The possible reason was that the probiotic complex promotes the development of the spleen, which leads to an increase in serum antibody levels. Abudabos et al. (2020) found that the addition of *B. licheniformis* and *B. subtilis* to diets significantly increased serum cholesterol, blood glucose, total protein and globulin levels in *Salmonella*-infected broilers. Similar results were obtained in this trial, where the addition of the probiotic complex resulted in a significant increase in both albumin and total protein in the serum of broiler. The increase in blood protein levels might be due to the fact that probiotics improves dietary protein utilization through its ability to inhibit pathogens growth, which reduces protein breakdown into nitrogen and increases the absorption of nutrients in the gut (Lee et al., 2013; Abd El-Moneim, 2017; Abd El-Moneim et al., 2020). In this study, blood glucose levels in the serum of broilers supplemented with 0.025% probiotic complex in the diet were found to be significantly higher than in the control and test broilers supplemented with 0.05% probiotic complex. This was inconsistent with the results reported by Al-Kassie et al. (2008) and Reuben et al. (2022), where the addition of probiotics reduced blood glucose levels in broilers. A dose-dependent relationship between blood glucose levels and the strains contained in probiotics had been previously reported (Samanya and Yamauchi, 2002). It is speculated that the reason for the inconsistency between this trial and previous results might be due to differences in the type of probiotics and the amount of additives, the exact reasons for which needed to be further investigated.

NH_3_, H_2_S, CH_4_ and CO_2_ were considered to be the most dangerous gases in the poultry organism (Gates et al., 2008). In this study, the addition of a probiotic complex was found to reduce NH_3_ and H_2_S emissions, reduced levels of *E. coli* and *Salmonella* and increase levels of *Lactobacillus* in broiler feces. Analysis of fecal microbiological and surface indicators showed positive and significant associations between *E. coli* and *Salmonella* levels and NH_3_ and H_2_S emissions. As previously reported by researchers, Misiukiewicz et al. (2021) concluded that fecal odor and NH_3_ emissions were associated with nutrient utilization and the gut microbial ecosystem. Along with *Pseudomonas*, *Citrobacter*, *Aeromonas* and *Salmonella*, *E. coli* was identified as the most promising H_2_S-producing bacterium (Maker and Washington, 1974). Jeong and Kim (2014) showed a decrease in *E. coli* and *Salmonella cecum* and an increase in *Lactobacillus* following the addition of *B. subtilis* preparations to broiler diets. The addition of a probiotic complex affected the fecal microbiota, increasing the number of beneficial bacteria and reducing the number of harmful bacteria in the broiler’s gut. When the number of *E. coli* and *Salmonella* decreases, the NH_3_ and H_2_S emissions in feces also decrease. And this test found a negative and significant relationship between *E. coli* and carcass yield. It also showed that with the reduction in *E. coli* numbers, the carcass yield of broilers is improved and more high-quality, healthy broiler meat was available. There was also a significant negative correlation between NH_3_ emissions and thymus index. As previously reported, growth performance of broilers was improved when the farm environment was improved (Jeni et al., 2021). Providing broilers with a better living environment will enable them to develop their thymus and thus achieve higher levels of immunity.

## Conclusion

Probiotic complexes: *B. subtilis*, *C. butyricum* and *E. faecalis* were added to AA+ male broiler diets to enhance growth performance and improve carcass performance. The addition of probiotic complex led to a reduction in NH_3_ and H_2_S emissions, a decrease in the number of *E. coli* and *Salmonella*, and an increase in *Lactobacillus* in the feces of AA+ male broilers. And based on the results of this trial, the recommended dose for use in on-farm production was 0.05%.

## Data availability statement

The original contributions presented in the study are included in the article/supplementary material, further inquiries can be directed to the corresponding authors.

## Ethics statement

The animal study was reviewed and approved by The Animal Conservation and Utilization Committee of the JZMU.

## Author contributions

All authors listed have made a substantial, direct, and intellectual contribution to the work and approved it for publication.

## Funding

This work was financed by the grants from the central government-guided local science and technology development fund projects (Grant No. 2021001). 2021 basic scientific research project of colleges and universities of Liaoning Provincial Department of Education (Grant No. LJKZ0801).

## Conflict of interest

The authors declare that the research was conducted in the absence of any commercial or financial relationships that could be construed as a potential conflict of interest.

## Publisher’s note

All claims expressed in this article are solely those of the authors and do not necessarily represent those of their affiliated organizations, or those of the publisher, the editors and the reviewers. Any product that may be evaluated in this article, or claim that may be made by its manufacturer, is not guaranteed or endorsed by the publisher.
